# Usages of Spark Framework with Different Machine Learning Algorithms

**DOI:** 10.1155/2021/1896953

**Published:** 2021-07-29

**Authors:** Mohamed Ali Mohamed, Ibrahim Mahmoud El-henawy, Ahmad Salah

**Affiliations:** Computer Science Department, Faculty of Computers and Informatics, Zagazig University, Zagazig, Egypt

## Abstract

Sensors, satellites, mobile devices, social media, e-commerce, and the Internet, among others, saturate us with data. The Internet of Things, in particular, enables massive amounts of data to be generated more quickly. The Internet of Things is a term that describes the process of connecting computers, smart devices, and other data-generating equipment to a network and transmitting data. As a result, data is produced and updated on a regular basis to reflect changes in all areas and activities. As a consequence of this exponential growth of data, a new term and idea known as big data have been coined. Big data is required to illuminate the relationships between things, forecast future trends, and provide more information to decision-makers. The major problem at present, however, is how to effectively collect and evaluate massive amounts of diverse and complicated data. In some sectors or applications, machine learning models are the most frequently utilized methods for interpreting and analyzing data and obtaining important information. On their own, traditional machine learning methods are unable to successfully handle large data problems. This article gives an introduction to Spark architecture as a platform that machine learning methods may utilize to address issues regarding the design and execution of large data systems. This article focuses on three machine learning types, including regression, classification, and clustering, and how they can be applied on top of the Spark platform.

## 1. Introduction

Big data is a term used to describe large, varied, and complex data collections that provide difficulties in terms of storing, processing, and presenting them for future processes or results. In 2021, Statista predicts that 74 zettabytes of data will be produced [1]. According to IDC, the total quantity of data in the globe will exceed 175 ZB by 2025, indicating a 61 percent compound annual growth rate [2]. Big data analytics is the process of analyzing massive amounts of data in order to discover hidden patterns and relationships. Big data research is at the forefront of current research and industry. Online transactions, emails, videos, audio files, pictures, click streams, logs, posts, web searches, medical records, social networking activities, scientific data, sensors, and cell phones and their applications all contribute to the creation of this data. They are stored in databases that grow in size and complexity quickly, making them difficult to gather, build, store, manage, distribute, analyze, and show using conventional database software tools. Big data has been defined in a number of ways by researchers, businesses, and individuals. The most common definitions of big data are Velocity, Volume, and Variety [3].

Apache Spark is a massively parallel in-memory processing solution that supports batch and stream data processing. Apache Spark's primary aim is to use in-memory computing to speed up batch data processing. For in-memory analytics, Spark has the potential to be 100 times faster than Hadoop MapReduce framework. Apache Spark's core engine provides fundamental cluster computing with in-memory functionality, such as fault recovery, memory management, job scheduling, and communication with databases [4].

The Spark application consists of five major components: worker nodes, cluster managers, tasks, driver programs, and executor processes. On a cluster, the Spark application works as a separate group of processes that are controlled by a SparkContext object. This object is the Spark entry point, and it is generated in a driver application, which is Spark's primary purpose. In cluster mode, SparkContext may interact with several cluster managers to ensure that the application has enough resources. YARN, Mesos, or Spark standalone clusters may be used as a cluster management system.

To allow the scalability of data algorithms at a high speed, the Spark engine provides an API for the main data abstraction used in programming such as the Resilient Distributed Dataset (RDD). RDD provides data analysis activities, such as transformation and manipulation of data, which may be utilized via the use of additional Spark libraries and tools. Spark's fundamental data abstraction is the RDD. It has a distributed collection of immutable objects, which may run in a parallel way. Because an RDD is immutable and cannot be altered once it is created, it is robust. As it is transmitted through many compute nodes in the cluster, an RDD is distributed. Every RDD is then divided into many divisions, each of which may be calculated on a separate node. This implies that the more divisions there are, the more parallelism there will be. In their driver programs, RDDs may be formed either by importing a dataset from another source or by parallelizing the existing collection of items [5].

Machine learning is a rapidly growing area of computer methods. They are designed to absorb knowledge from their environment to imitate human intelligence. Machine learning has been used in a wide range of fields, including computer vision, entertainment, and biology. A machine learning algorithm is a computer software that performs tasks using input data. The goal of machine learning is to mimic how people learn to understand sensory (input) data to accomplish a job [6].

Supervised, unsupervised, semisupervised, reinforcement, evolutionary, and deep learning are all examples of machine learning techniques. These techniques are used to categorize the information gathered. We will focus on the two most often used types of machine learning techniques, supervised and unsupervised learning methods [7].

Supervised learning: algorithms respond appropriately to all potential inputs when given a training set of examples with suitable objectives. Supervised Learning is another name for exemplar-based learning. Classification and regression are two examples of Supervised Learning.

The main two tasks of supervised learning are classification and regression. Classification is the process of grouping data into predetermined groups or classes prior to analysis. Classification is the process of classifying objects, data, or ideas based on their shared features [8]. Regression is a method that uses example data to infer relationships between continuous inputs and outputs to generate predictions for fresh inputs [9].

Unsupervised learning: unsupervised learning is methods looking for similarities between input data and classify the data accordingly. Additionally, this is referred to as an estimation of the density. There are no correct answers or objectives in unsupervised learning. Clustering is a technique that is used in unsupervised learning. Clustering: it is the process of finding a structure or pattern within a group of unlabeled datasets. Clustering techniques split a given dataset into K clusters in such a manner that data points within each cluster are linked to one another but are different from data points in other clusters [10].

As per the new requirements for business analysis and the massive amounts of data generated by all smart devices, sensors, satellites, social media, and other sources, as well as by new discoveries in various multidisciplinary sciences, the currently applied methods of standalone data processing, such as machine learning algorithms, face numerous challenges to meet these demands. All of these issues may be summed up in a single phrase called big data or a set of attributes such as volume, velocity, and variety. All of these issues have an impact on machine learning's ability to cope with the new age of big data. Numerous platforms have been developed to address these problems, including Hadoop and Spark. We present Spark framework in this article as one of the most applicable solutions to large data problems using machine learning methods.

In this study, we show a range of approaches and tactics for using machine learning techniques on top of the Spark architecture, demonstrating how machine learning methods can be utilized to deal with the problems of big data. The following is the structure of this essay: Section 2 goes over different applications and models of Spark and regression methods for real-world problems.  Section 3 shows the applied solutions of Spark and machine learning classification methods. In Section 4, Spark and unsupervised clustering machine learning algorithms are shown to be successful solutions in many cases. Finally, the paper is concluded in Section 5.

## 2. Regression Algorithms Using Spark

Radial basis function (RBF), general regression neural network (GRNN), wavelet neural network (WNN), group method of data handling (GMDH), and multilayer perceptron (MLP) are all prominent neural network designs for prediction. GRNN is superior to the other two designs, since it entails single-pass learning and provides fairly acceptable outcomes. Although GRNN employs single-pass learning, it was incapable of handling large datasets due to the need for a pattern layer, which is capable of retaining all cluster centers after the clustering of all samples. As a consequence, GRNN++, a hybrid architecture, was introduced that scales GRNN for huge data sets by invoking a parallel distributed version of K-means++ dubbed K-means|| in the pattern layer of GRNN. The proposed solution architecture was built using Apache Spark's distributed parallel computing architecture in conjunction with HDFS. GRNN++ performance was evaluated using a 613 MB gas sensor dataset with tenfold cross-validation. The suggested GRNN++ algorithm generates very little mean squared error (MSE). This paper's main objective was to provide a distributed and parallel implementation of the conventional GRNN [11].

Predicting the magnitude of earthquakes is a difficult issue that has been extensively researched throughout the past decades. There are many statistical, geophysical, and machine learning methods in the literature, but none of them provide especially acceptable results. Recent years have seen the emergence of sophisticated computing methods for analyzing big data, enabling the study of enormous datasets. These novel techniques use physical resources, such as cloud-based systems. California is well known for being among the world's greatest seismic activity, and many data are accessible.

California is well known for having among of the world's greatest seismic activity, and many data are accessible. Until now, conventional machine learning techniques have been used to forecast earthquakes. However, the examined datasets were usually no more than a few MB in size. A 1GB library was created for this purpose, including occurrences from 1970 to 2017. Four regressors (linear models, gradient boosting machine, deep learning, and random forest) were used individually and in ensembles to forecast the maximum magnitude over the next seven days. Due to the large computing resources needed, it is essential to use big data technologies and infrastructure. This method utilized the Spark distributed computing framework and the R language's H_2_O package for cluster computing. Finally, Amazon cloud computing infrastructures were used. Stacking-based ensemble learning was used, which produced relative errors of around 10% and absolute errors of about 0.5. Techniques based on trees performed better, and, in general, these methods achieved lower regression errors. The Apache Spark framework, the R language's H_2_O library, and Amazon cloud infrastructure were utilized, with extremely promising results [12].

In recent years, high performance computing (HPC) has become a popular subject. A supercomputer is one of the world's most popular HPC products. However, the conventional supercomputer is no longer the dominant computing platform, and its availability has shifted significantly as a result of its high cost and limited accessibility. Thus, a low-cost and easily accessible HPC system is needed. There are three kinds of contemporary HPC systems that are more accessible and affordable than conventional supercomputers. Grid, cloud, and cluster computing are all examples of these. Cluster computing was developed to overcome supercomputers' dominance. Cluster computing is capable of resolving issues that cannot be successfully addressed by supercomputers. Typically, data mining is performed on a stand-alone computer. However, depending on the size of the dataset, mining the data on a single PC may take many seconds, minutes, hours, or even days. A solution is required to increase the efficiency of mining operations, particularly in terms of processing time.

A data mining method was developed in a cluster environment to speed up the mining process in the research. The performance of cluster computing was evaluated by comparing it to different node counts and to standalone computing. The research's case study involved forecasting flight delays using a linear regression method. All simulations were run inside a virtual environment. The result indicated that, by combining five PCs with identical specifications into a cluster environment, the performance of computing was increased by up to 39.76 percent as compared to a standalone environment. By adding more nodes to the cluster, the process might be substantially accelerated [13].

Spark provides over 180 configuration options to ensure that periodic tasks run efficiently. Setting these settings has a significant effect on the overall performance, since they were initially set to default values when Spark was deployed. By selecting the optimal setup, Spark's performance might be substantially boosted. However, since the ideal Spark configuration is application-specific, implementing a single configuration or changing a single parameter results in suboptimal performance.

Not only are the Spark setup options simple, but they also interact in complicated ways. Additionally, when the input data collection is large, each run of the Spark program takes a significant amount of time. These problems exacerbate the difficulty of forecasting the performance of Spark applications in any arrangement. Performance models are often a useful method to assist in resolving problems, since they can anticipate much more quickly than an approach that needs the program to be executed.

This research offered a method based on AdaBoost projective sampling for efficiently and correctly forecasting the performance of a given application on a particular Spark setup. To reduce modelling overhead, traditional projective sampling was used. In machine learning, the AdaBoost algorithm is a type of ensemble learning. It creates and combines many learners to complete learning tasks and has a high prediction accuracy when dealing with complex high-dimensional problems. The stage-level performance prediction model was trained using data from the actual Spark system. The model accepted the Spark settings as input and generated a forecast of the model's performance. The method was evaluated using six standard Spark benchmarks, each with five distinct input datasets. The findings indicated that the average error of the model built using the proposed method was just 9.02 percent, which was much less than the average error of the current approaches [14].

This study discussed a technique for predicting time series data using deep learning. The H2O big data analysis framework's deep feed forward neural network was utilized in conjunction with the Apache Spark platform for distributed computing. Due to the fact that H_2_O does not support multistep regression, a general-purpose technique was offered to be utilized for prediction horizons of any length, where *h* is the number of future values to be predicted. The approach entails subdividing the issue into **h** forecasting subproblems, where *h* denotes the number of samples to be forecasted concurrently. Thus, the optimal prediction model for each subproblem may be determined, enabling parallelization and adaptability to the big data environment to be simpler. Additionally, a grid search was used to determine the deep learning-based approach's optimum hyperparameters. The authors provide results from a real-world dataset of energy usage in Spain from 2007 to 2016, using a ten-minute frequency sampling rate. The accuracy and runtime were evaluated in relation to the computer resources and dataset size, with the goal of achieving a mean relative error of less than 2%. The method's scalability was evaluated in terms of time series length and number of execution threads, and it demonstrated linear scalability and excellent performance for distributed computing. Finally, the methodology's accuracy and scalability have been compared to those of other previously published methods. Deep learning has been shown to be one of the most suitable techniques for processing large data time series, alongside decision trees, in terms of scalability and accuracy [15].

## 3. Classification Algorithms Using Spark

SARS-CoV-2 (COVID-19) spread quickly across the globe, eventually resulting in a worldwide epidemic. It has had a debilitating impact on public health. Thus, it is critical to identify positive instances as soon as they are feasible to treat the affected individuals promptly. Chest Computed Tomography (CT) is one of the diagnostic tools for 2019-nCoV acute respiratory illness. Advanced deep learning methods in conjunction with radiological imaging may aid in the accurate identification of the new coronavirus. Additionally, it may help in overcoming the tough circumstances created by a shortage of medical expertise and specialist physicians in distant areas. This research demonstrated how to use Apache Spark and KerasTensorFlow in conjunction with the Logistic Regression method to identify COVID-19 in chest CT scans automatically, utilizing the Convolutional Neural Network (CNN) models VGG16, VGG19, and Xception. The model correctly classified VGG16, VGG19, and Xception with an accuracy of 85.64, 84.25, and 82.87 percent, respectively. This approach enabled physicians to get a precise and definite picture of the presence or absence of COVID-19 in chest CT scans, enabling Moroccan medical professionals to utilize its sophisticated capabilities as a diagnostic method in medical imaging [16].

Data security refers to the process of safeguarding data from security breaches and hackers. The intrusion detection program is a software framework that continuously monitors and analyzes network data to discover assaults using conventional methods. These conventional infiltration methods are very efficient when dealing with tiny amounts of data. However, when the same methods are applied to large data, the process of evaluating the data characteristics becomes inefficient, necessitating the usage of big data platform such as Hadoop, Apache Spark, and Flink to build a contemporary intrusion detection system (IDS). The architecture of Apache Spark and a classification algorithm-based IDS was described in this study, along with a technique for picking features from network security event data using Chi-square. SGD was used to assess the performance of Logistic Regression, Decision Trees, and SVMs in the creation of an Apache Spark-based IDS using AUROC and AUPR as metrics. Additionally, the training and testing times for each method were set through all of the experiments using the NSL-KDD dataset. The dataset is comprised of the files KDDTrain+.txt and KDDTest+.txt. The dataset contains 125,973 training and 22,544 testing instances, and all instances in the training and testing files are utilized. There are 41 characteristics and a single label for the class.

The performance and accuracy of various algorithms are determined and utilized by IDS in a big data platform that analyzes huge datasets. To determine if the data is normal or manipulated, decision trees, SVMs, and Logistic Regression are employed. The findings indicated that the decision tree achieved a higher level of accuracy and logistic regression required less time than other methods [17].

According to the development of sensors integrated into mobile devices, the analysis of everyday human actions has become more accessible and widespread. Numerous applications, such as health analysis, fitness monitoring, and user-adaptive systems, have been created by delving deeply into complicated human behaviors. The purpose of this article is to provide a system for monitoring and recognizing the activities of older people using the Apache Spark platform for big data processing technology. To identify human behavior in the suggested framework, several classification methods such as random forest classifier, decision tree, and logistic regression, and from Apache Spark's ML machine learning package, were utilized. Two well-known datasets, WISDM, smartphone accelerometers, and KAGGLE-UCI, are utilized to validate the outputs of the proposed system. The performance of classification-based methods for recognizing human activities is assessed in terms of accuracy, F1-score, training time, and testing time.

In the tests, it was discovered that a 70–30 dividing ratio, which allocates 70 percent of the data to the training phase and 30 percent to the testing phase, was the most convenient configuration for dealing with a range of data. Thus, this prevents the overtraining problem at the dataset, while still ensuring that it is sufficiently trained for testing purposes. The examination of the results revealed that Logistic Regression with Cross-Fold Validation performed superior to other methods in terms of F1-score and accuracy. When it was evaluated against WISDM Time series data from the human activity recognition accelerometer sensor, an accuracy of 72.10 percent was obtained. It achieved a maximum accuracy of 91.02 percent when evaluated against time series data from the KAGGLE-UCI Human Activity Recognition Accelerometer-Sensor [18].

This study identifies VPN network traffic using time-related characteristics extracted from Apache Spark and artificial neural networks. Today's Internet traffic is encrypted utilizing VPN/Non-VPN protocols. This circumstance precludes the use of traditional deep packet inspection techniques that analyze packet payloads. MATLAB 2019b was used to carry out this study, since the growing demand for VPN networks has triggered the development of technology. The suggested approach eliminates the redundant processing and flooding associated with conventional VPN network traffic categorization. As the proposed system is trained on 80% of the dataset, only 20% is retained for testing and validation, using 10-fold cross-validation and 50 training epochs. According to the authors, this was the first research that introduced and used artificial neural networks with the Apache spark engine to classify VPN network traffic flow. VPN categorization accuracy is 96.76 percent, while utilizing ANN with Apache Spark Engine. The suggested approach has a classification accuracy of 92.56 percent for non-VPNs. This research established that a method based on the CIC-Darknet2020 standard for packet-level encrypted traffic categorization could not include packet header information since it enables high-precision mapping of a packet to a particular application. 96.76 percent of all packets in the sample could be attributed to an application when just non-VPN traffic was included [19].

As data processing services have grown in popularity, it is fairly uncommon for a computer system to have many tenants that share the same computer resources, resulting in performance anomalies caused by resource contention, faults, workload unpredictable nature, software bugs, and a variety of other main causes. For example, while application tasks may show variations in execution time as a result of varying dataset sizes, the inconstancy of the platform's scheduling problems, interruptions from other programs, and software assertions from other users may lead to unusually long running times viewed as abnormal by the end users.

Performance abnormalities detected late and manually resolved in big data and cloud computing platforms may result in infractions of performance and financial penalties. To address this issue, an artificial neural network-based method for anomaly detection was created and adapted for the Apache Spark platform, which utilizes in-memory processing. While Apache Spark has gained widespread adoption in business because of its high performance and flexibility, there is currently a lack of comprehensive methods for detecting performance anomalies on this platform. The proposed technique was based on artificial neural networks for rapidly sifting through the data log in Spark and the metrics of monitoring the operating system to correctly identify and categorize abnormal behaviors depending on the features of the Spark resilient distributed datasets. The suggested approach is compared to three well-known machine learning methods: support vector machine, decision trees, and closest neighbor, as well as four versions that take into account various monitoring datasets. As per the findings, it is demonstrated that the suggested approach outperforms existing methods, usually reaching 98–99 percent F-scores and providing much better accuracy in detecting both the period during which anomalies happened and their kind than other strategies [5].

## 4. Clustering Algorithms Using Spark

In general, current techniques for triangle counting are incapable of handling large graphs. This work contributes by presenting an adequate and flexible method for counting triangles and calculating clustering coefficients on Apache Spark framework to address the problems and obstacles associated with large graphs. CCFinder was developed as a straightforward, nevertheless effective, and scalable method for computing the coefficients of clustering on a local and global scale, respectively, based on MapReduce paradigm and the Spark platform. While Hadoop has been the most well-known open-source MapReduce implementation, the proposed method was built on Apache Spark infrastructure because of its superior performance and optimization capabilities. Spark has a storage called Resilient Distributed Datasets (RDDs), which allows users to preserve data across jobs and provides fault tolerance even without the requirement for replication. According to its major capability, the method caches the reused data structure into distributed memory, resulting in a significant speed boost. It is worth noting that the method was not entirely dependent on the functions of Map and Reduce, and it also used cogroup operator, which is one of Spark capabilities. Cogroup operator connects two RDDs to each other on the basis of their keys, creating a new RDD with a shared key and two values that match the inputs of RDDs.

The method was unique in the following ways. An effective data structure named (Filtered Ordered Neighbor List) FONL was created, cached in Spark's distributed memory, and then utilized in the subsequent stages of CCFinder. Each item inside the FONL includes just the information needed that substantially lowers storage needs, maximizes fine-grained parallelism, and enhances load balancing. Even though the FONL data structure needs a little amount of memory, if it cannot be completely cached into distributed memory, the available hard drive space will be utilized to retain the portions of the data structure that do not fit in memory. CCFinder was able to quickly determine the triangle count in a dedicated graph precisely by using the FONL. The method of triangle counting is modified to calculate the clustering coefficient, including the degree information about the vertices in the proper locations.

CCFinder begins by constructing a list of neighbors to every vertex. The FONL is constructed from the neighbor listings that have been generated. Each FONL item consists of a key-value pair, with the key component being a distinctive integer denoting a graph vertex. To stay away from performing additional calculations (when counting triangles), only neighbors of the related vertex (indicated by the key part) with a degree higher than the level of the key vertex are included in the component. Another data structure is also produced from the FONL to be combined with FONL to locate the triangles. CCFinder calculates clustering coefficients using the Spark abstraction. It produces less scrambled data and performs quicker than current similar techniques during execution, since it creates as few things as feasible and reuses cached data. To demonstrate the suggested method's efficiency and scalability, it was built on top of a Spark cluster with 60 CPU cores and 500GB of memory and tested on many common real-world graph datasets. Directed and undirected graphs were considered. Twitter, which has approximately 55.5 billion triangles, was anticipated to be the most difficult of these graphs. Scalability was found to be adequate over a range of core counts. Additionally, the proposed method may be more efficient than other distributed implementations such NodeIterator++ based on MapReduce and Cohen's method [20].

Every day, as technology advances, a massive amount of data containing valuable information, dubbed Big Data, is produced. To handle such massive amounts of data, big data platforms such as Hadoop, MapReduce, Apache Spark, and others are required. Apache Spark, for example, outperforms traditional frameworks like Hadoop MapReduce by up to 100 times. Scalable Machine Learning techniques are needed to overcome space and temporal constraints in the processing and interpretation of this data. Due to their cheap processing needs, partitional clustering methods are frequently used by academics for clustering big datasets. Thus, the research examined the design and development of a technique for partitional clustering using Apache Spark. The research presented a partitional-based clustering method called Scalable Random Sampling with Iterative Optimization Fuzzy c-Means algorithm (SRSIO-FCM) that was built on Apache Spark to address the difficulties of Big Data Clustering. The efficacy of SRSIO-FCM is shown via comparison with a suggested scaled version of the Literal Fuzzy c-Means (LFCM) algorithm dubbed SLFCM built on Apache Spark. The comparison findings were expressed in terms of the F-value measure's ARI, objective function, run-time, and scalability. The findings demonstrate SRSIO-enormous FCM's potential for Big Data clustering [21].

Fusion of sensor data is a crucial component of active perception in autonomous robots in order for them to see their environment properly, make appropriate adjustments to improve their understanding of it, and provide actionable insights. Despite the existence of a large body of research in this area, just a few publications focus exclusively on unsupervised machine learning, which is becoming more essential in unlabeled, unknown environments.

The study offered an expandable self-organizing neural design for environmental perception based on unsupervised machine learning and multimodal fusion. The neural architecture, inspired by its biological equivalent, is composed of topographic maps for each modality and includes a new scalable self-organization mechanism capable of handling large volumes of high-velocity sensor input. Cross-modal connections are utilized to express cooccurrence relationships across modalities, and the reverse Hebbian projection is employed for multimodal representation. Apache Hadoop and Apache Spark created this scalable fusion method based on unsupervised machine learning. It was tested on a large multimodal dataset of human activity sensors to show efficient representation and multimodal fusion for active perception. Additionally, the good cluster quality findings indicate that multimodal fusion improved the outcomes [22].

## 5. Conclusion

This article has given a thorough examination of the use of the Spark framework in conjunction with machine learning methods. The goal of this review is to demonstrate the value of combining Spark, one of the most efficient distribution system frameworks, with machine learning methods to adapt to the requirements of large data systems. There were three kinds of machine learning techniques: supervised machine learning regression, supervised machine learning classification, and unsupervised machine learning clustering, all of which were used with the Spark framework in various areas.

## Data Availability

No data were used to support this study.
